# Improved ergonomic layout design of metro control center based on virtual simulation technology and genetic algorithm

**DOI:** 10.1038/s41598-023-47671-y

**Published:** 2023-12-05

**Authors:** Hanzhao Qiu, Weining Fang, Jianxin Wang, Yueyuan Chen

**Affiliations:** 1 School of Mechanical, Electronic and Control Engineering, Beijing Jiaotong University, Beijing, 100044 China; 2 China Nuclear Power Engineering Co., Ltd., Beijing, China

**Keywords:** Mechanical engineering, Computer science

## Abstract

For the existing control center layout methods do not have targeted measures to implement human factors requirements, this paper puts forward a specific human factors design method for metro control center layout. This method covers the overall process of ergonomic standards and actual engineering requirements in personal space determination, overall location design, and personnel post layout. It introduces discrete personnel simulation to assist in physical control center layout design for the controllers’ safety. To make the posts between the unified lines and the connections between the lines more reasonable, an improved genetic algorithm is used to solve the Systematic Layout Planning. This design flow and algorithm can be quickly adapted according to the project's actual needs and the application of human factors guidelines. This method is also suitable for the layout planning and design of similar large spaces, large numbers of people, and complex associated control centers.

## Introduction

The metro control center is a command platform that integrates line operation control, network coordination and management, and network emergency command. As metro construction in various countries gradually enters the stage of large-scale and networked operation development, the relationship between the control center lines has become closer, and higher requirements have been placed on operation and management. In order to ensure its effective use of line operation monitoring and command functions, reasonable and effective design of the metro control center is crucially important. Unreasonable internal design of the control center will lead to confusions in the allocation and coordination of human resources. In that case, it will affect the safe operation of the entire city's subway. Once an accident occurs, it will have immeasurable and severe consequences^1,2^. In the control center's design process, the application of more human factors or human factors engineering-related knowledge can eliminate or reduce the occurrence of human errors and improve the efficiency of human work^3^. Swain and Guttman's research found that the introduction of human factors engineering design standards in workstation design reduces human error probability by 2 to 10 times^4^. In order to ensure the safety and efficiency of the metro network, and enhance the response to major incidents and emergencies, the control center layout design that meets the requirements of human factors engineering is imperative.

In the control center's design, providing a safe, comfortable, and well-functioning environment for the control center personnel is essential to the performance of the control center function^5^. Takashi Naito pointed out that, unlike general office design, the control center's design needs to be combined with a human-centered design method to achieve a balance between the comfort and functionality of the control center. The design requirements mainly include the operational tasks of the control center under normal and critical conditions, Operational organization analysis, human factors standards related to control center design, and human factors engineering-related design requirements^6^. Leonardo Quintana et al. studied the visualization of human factors engineering to guide the design of petrochemical process control centers. It includes optimizing workstation design, control room layout, building layout, lighting, acoustics, and environmental design, which improved the control center's productivity and improved the control room's performance^7^. Skřehot et al. regarding the control center and its operators as a complex overall work system for design research, designed a methodology tool for implementing the human-centered principle, defined 208 human engineering parameters, and 12 areas division, including layout solutions, sanitary conditions, microclimate conditions, acoustic conditions, visual conditions, workstations, large display screens. These parameters can be used in practice as a supplementary professional for the safety management system, and occupational safety and health audits tools^8^. Although the above-mentioned work can solve the layout problem to a certain extent, the corresponding labor and time costs would increase significantly in the context of increased design elements.

There are some relevant international standards, regulations, or guidelines specifically for the human factors engineering design of control centers^9^. An important example is the ISO 11064 series of standards^10^, whose goal is to eliminate or minimize human errors in control center design^11,12^. As the guiding standard for human factors engineering design of control centers, ISO 11064 series standards are widely used in the design of control centers in various fields. Francisco Duarte et al. studied the design of the deep-water platform control center based on human factors engineering intervention. Some principles of the ISO 11064 standard, such as scenario analysis, work simulation, and user participation, were run through the entire design process^13^. Skramstad, Torbjørn et al. used a combination of semi-structured interviews and online surveys to study the application of the ISO 11064 standard in the design of the Norwegian petroleum industry control center and affirmed the activeness of the ISO standard in the safe operation of the control center and the design of improving working conditions^14,15^. Existing research has proved the guiding role of standards in the design of control centers in engineering practice, but in the design goal of clearer professional subdivision and a higher degree of connection between different work units, further consideration of the connection between operating units is required.

Due to the disadvantages of the existing design methods, such as high labor cost and time-consuming, most actual design of the subway control centers layout focuses on principles, engineering experience or existing problems^16^. There is no targeted implementation of normative principles and human factors standards, resulting in unreasonable layouts in the control center design, causing health injuries to personnel, and low job satisfaction^17^. Based on the analysis and research of ISO 11064 series standards and other standards for control room design, this paper proposes a human factor layout design method that meets the operation requirements of metro control center, which focuses on the post relevance. The successful application of this method in the Shanghai Metro Control Center verifies the effectiveness of this method. The presented method covers the overall process of ergonomic standards and actual engineering requirements in personal space determination, overall location design, and personnel post layout. This design flow and algorithm can be quickly adapted according to the project's actual needs and the application of human factors guidelines.

The following sections describe this method and its application in the case study of the Shanghai Metro Control Center. In Section “Outline of this study and methodology”, the overall process of the method is described. Section “Physical layout of positions based on simulation” proposes a preliminary control center layout scheme based on human mobility. Section “Optimization for the ergonomics layout model” puts forward the post layout optimization method of the control center. In the fifth part, the optimal layout model is solved by an improved genetic algorithm. The results and limitations of this method are discussed in Section “Discussion”. Finally, some conclusions are drawn in Section “Conclusion”.

## Outline of this study and methodology

The methodological approach of this study included a research process of the system “operator–metro control center”: operator (anthropometric and kinesiological research, workflow relevance analysis); control center (ergonomic design of control desk, and ergonomic layout optimization).

**Figure 1:**
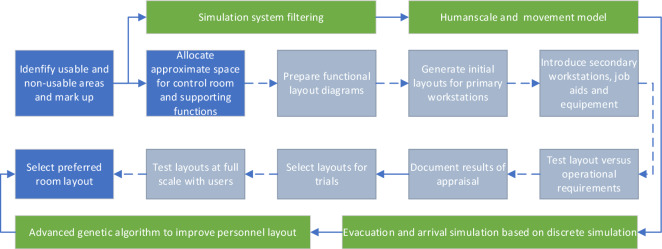
General procedure for control room layout compared with traditional layout process. The blue background box represents the major steps of the traditional layout process, the gray background box represents the detailed steps of the traditional layout process, and the green background box represents the method flow proposed in this article.

The overall design (Fig. 1) begins with the selection of anthropometric methods, selection and determination of human factors standards, and preliminary selection of physical layout schemes. In the follow-up, it is necessary to carry out job layout constraints, target determination, and layout algorithm solving. It will be checked and verified with the design target to determine the final layout. The determination of the physical location plan was mainly through subject-matter experts’ design to form the first draft. These combined with the physical location constraints in the human factor standard and the simulation results of personnel mobility. In the stage of assigning positions, combining the relevance between positions, iterations are carried out through artificial intelligence algorithms to obtain the optimal solution quickly.

These difficulties in the process need to be resolved to enable the workflow to progress smoothly. First, in the determination of personal layout space, in the past, it was easy to have too many redundant or too little equipment, which requires more scientific and quantitative determination results. Based on the collection of basic task requirements, the personal space requirements are determined by simulating the human body model's action. Secondly, in the physical layout, the previous process is relatively subjective, and the efficiency of personnel flow is very low when responding to the situation of evacuation or handover. A more reasonable and safer physical layout could be determined through the discrete simulation of personnel moving. In the final personnel arrangement stage, problems such as the inconvenience of poor communication are prone to occur. Then the specific personnel arrangement plan is determined through a genetic algorithm. All the processes dramatically reduced the time and economic cost of physical model manufacturing and on-site user evaluation.

## Physical layout of positions based on simulation

### Framework for initial ergonomic layout analysis based on physical limitations

The metro control center is a multi-level dispatching command center. The rationality of the space design for the dispatcher's operation activities is mainly reflected in the rationality of the console size design^16^. According to the requirements of human factors engineering, when designing the console, human abilities, physiological limits, and psychological needs should be considered. The design data of the console should conform to the characteristics of the user's group.

The design of the console should be based on work analysis. Each work and its results and the requirements of the corresponding work area are the basis for the console design^18^. Besides, the shape of the console is affected by the characteristics of the input device, the use of communication equipment and display devices, the frequency of use, and the interaction with other positions^19^. The human adaptability design of the console should follow the following steps. Firstly, analyze the responsibilities of each position and the requirements of the display and control equipment used, such as the number of users, visual requirements, and communication with other positions. And then list the display and control equipment (displays, driving dispatchers), communication equipment and other work equipment that are required to be used by each post, as well as detailed descriptions of the equipment, such as screen size, display, and control equipment model, shape and size, and Installation method, etc. According to the work task, and work posture, carry out the layout design, structure size design, and maintenance workspace design of the console display and control device. Finally, the overall geometric sizes of the console are determined.

After the research on the space requirements of the dispatcher's console of each post in the prework, combined with the control center's personnel requirements for operation control, the consoles of different positions can be placed in the control center to obtain the physical layout. Since there are many people in the control center, the comprehensive analysis of the mobility of personnel gives reference to the optimal layout.

### Ergonomic limitations of physical space and layout alternatives

**Figure 2:**
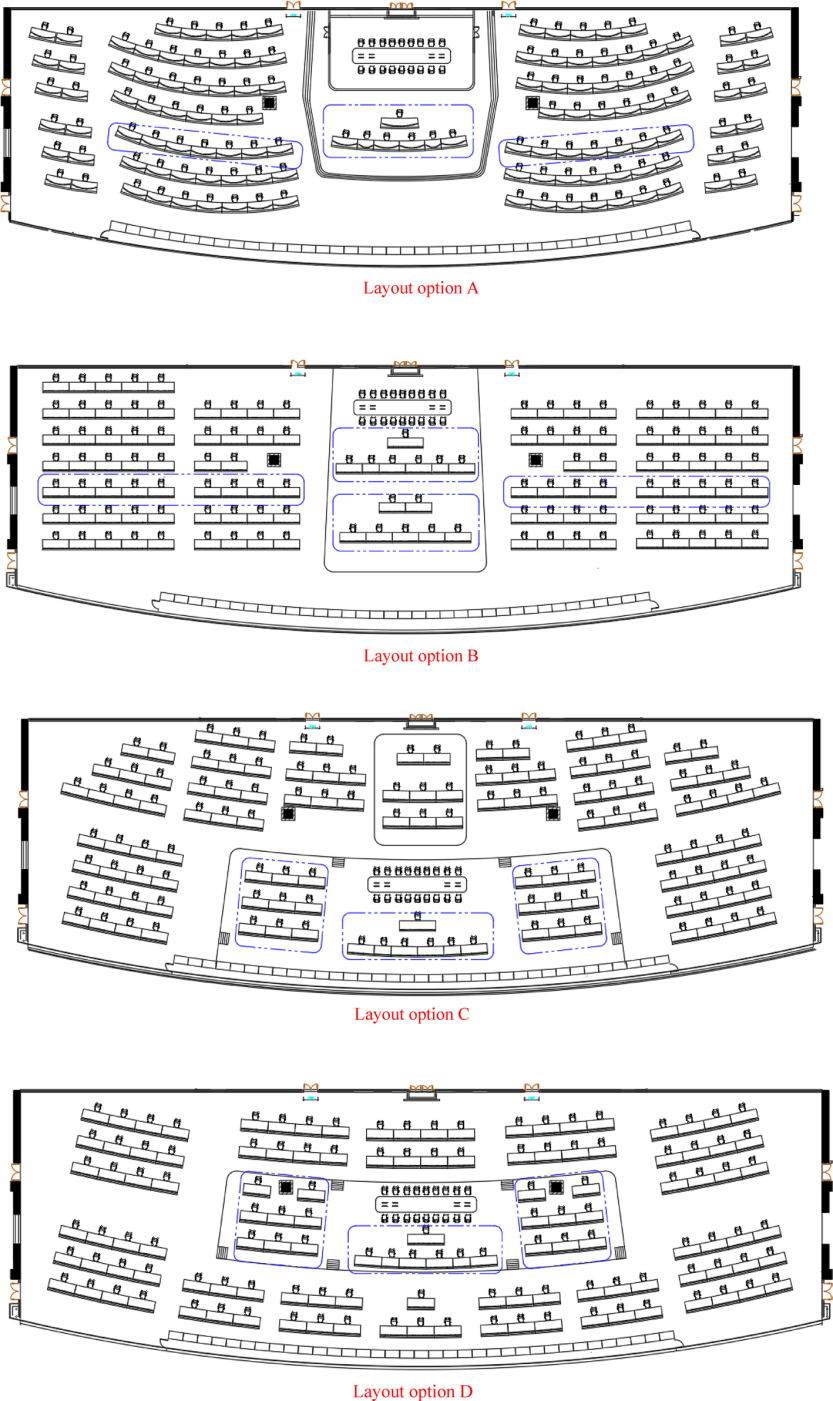
Four layout examples.

With the need to arrange a total of 14 lines in the Shanghai Metro Control Center, summarizing ISO11064 series standards, Chinese national standards and industry standards, the main restrictions are obtained^10,20–25^. In the first phase, a total of 14 lines needs to be introduced. And all of the 123 dispatching stations and conference tables need to be arranged. For the requirements of the layout of the metro control center as well as the actual building restrictions and user constraints, four general layout schemes of the Shanghai Metro Control Center were designed, as shown in Fig. 2.

Layout option A places the COCC and conference table centrally on the steps at the back of the control center. Network video monitoring and network power dispatch are respectively arranged in the middle of the left and right sides of the steps, while the other positions are OCC traffic dispatch and comprehensive dispatch positions. The dispatch desk is arranged in an inner arc layout, symmetrically inclined to the left and right. According to the different number of traffic dispatch positions required for the line, a 2 + 6 (7) layout is adopted.

Layout option B concentrates the COCC, regional dispatch chief, and conference table on the steps behind the control center. Network video monitoring and network power dispatch are respectively arranged in the middle of the left and right sides of the steps, while the other positions are OCC traffic dispatch and comprehensive dispatch positions. The control consoles are arranged in a straight line and placed horizontally. The control consoles in the OCC area, network video monitoring, and network power dispatch area are arranged in a 5 + 4 manner, except for two control consoles placed in the column area.

Layout option C places the COCC and conference table centrally in the central position in front of the control center. Network video monitoring and network power dispatch are respectively arranged on the left and right sides of the COCC area, located above the steps in the front of the control center. The remaining positions are OCC traffic dispatch and comprehensive dispatch positions. The dispatch desk is arranged in a straight line, symmetrically inclined on the left and right sides, arranged according to the line, with 3 to 4 positions placed in a group.

Layout option D places the COCC and conference table in the central position of the control center, with network video monitoring and network power dispatch respectively arranged on the left and right sides of the COCC area, located above the steps in the center of the control center. The surrounding positions are OCC traffic dispatch, comprehensive dispatch, and regional dispatch chief positions. The dispatch desk is arranged in a straight line, symmetrically inclined on the left and right sides, arranged according to the line, with 3 to 4 positions placed in a group.

### Analysis of personnel mobility

The layout design of the metro control center needs to consider the influence of multiple factors, including the overall structural size of the control hall, the location of the exit channel, the relationship between the consoles, the communication needs between staff, visitors, and the shifting process The patency, arrival, and evacuation of posts in emergencies, the number of personnel and their dynamic changes^17^.

This study used the pedestrian micro-simulation software LEGION based on discrete simulation to evaluate the overall initial layout of the control center. Discrete simulation method of human behavior in the process of simulation model construction and analysis, a single individual in the crowd was regarded as the research object. More attention was paid to the behavior characteristics of the individual and the relationship between them. The simulation needed to consider the different parameters and behavior characteristics of the individuals in the crowd, genuinely reflect the interaction between the people and the interaction between the people and the surrounding obstacles^26^. Furthermore, it was necessary to simulate the passenger flow conditions under normal and emergency states to simulate individuals' behavior characteristics in the evacuation and shift of the metro control center. Therefore, the simulation analysis of the overall layout of the control center is divided into two aspects. First, the control center's dispatcher needs to enter the control center's designated position at the same time when the shift is transferred. To effectively improve the efficiency of the control center's shift, find the time when the personnel is in place. A short layout plan without a high density of personnel requires a simulation analysis of personnel arrival; secondly, an analysis of personnel's safe evacuation in the control center is considered when a critical situation occurs. Legion has received good impressions in three Olympic Games after 2000 and London’s Olympic bid projects^27^. In addition, it has some applications in rail transit, such as the New York subway planning, and Hong Kong subway station planning^28^. Therefore, this paper uses Legion to conduct the simulation.

The method based on discrete simulation experiments is adopted to improve the efficiency of safe evacuation, reduce the shift work time as the overall layout plan selection idea, and compare and screen different layout plans. The simulation analysis steps of evacuation and shift of the Shanghai Metro Control Center are as follows.

- Step 1. Build the overall layout scheme model of the control center.
  According to the four general layout schemes of the control center designed above, the two-dimensional plane layout CAD drawing is drawn, and the simulation scene is established for the next step.
- Step 2. Determine the attribute parameters of the dispatcher in the control center and run the simulation model.
  Firstly, set the simulation run time. Then, input the dispatcher attribute parameters, such as baggage carrying condition, and speed parameters calculated based on the dispatchers' gender and age ratio. Next, determine the personnel walking route. Finally, establish the analysis line and run the simulation. The basic information of the Shanghai Metro dispatching personnel is collected by means of questionnaire survey. They are analyzed and processed as the input of personnel attribute parameters. The data are shown in Tables 1 and 2.

**Table 1:** Dimension data of the Shanghai metro control center.

Total area (m^2^)	Number of dispatchers (person)	Standard dispatching desk area (m^2^)	General dispatcher 1 area (m^2^)	Public dispatching 2 area (m^2^)
2150	139	1.1 × 2.8	1.1 × 3.8	1.1 × 1.8

**Table 2:** Gender and age ratio of dispatchers.

Gender	Male	Male	Male	Female	Female	Female
Age	18–25	26–35	36–45	18–25	26–35	36–45
Ratio	38.85%	41.73%	6.47%	5.76%	6.47%	0.72%
  Referring to the relationship between personnel walking speed, gender and age obtained from experiments of the International Maritime Organization (IMO), combined with investigated data above, the speed data of dispatchers of different gender and age distribution can be obtained as shown in Table 3.

**Table 3:** Personnel walking speed distribution.

Gender	Male	Male	Male	Female	Female	Female
Age	18–25	26–35	36–45	18–25	26–35	36–45
Average speed (m/s)	1.6 (77.78%) and 1.5 (22.22%)	1.5 (79.31%) and 1.4 (20.69%)	1.4 (100%)	1.3 (50%) and 1.4 (50%)	1.3 (44.44%) and 1.2 (55.56%)	1.1 (100%)
- Step 3. Simulation analysis of overall layout scheme of control center.
  The simulation output analysis indicators include evacuation time, personnel quantity statistics, cumulative high density map, cumulative maximum density map, etc. In order to reduce accidental errors, each layout option of the metro control center runs simulations 15 times independently, eliminating the instances where the evacuees are not completely evacuated. The 3δ criterion is used to eliminate the simulation data whose absolute value of the difference from the mean value of the simulation time is higher than three times the standard deviation. The average evacuation time of four layout schemes are shown in Fig. 3. It can be seen that among the four layout schemes, the average evacuation time of scheme A is the shortest, the evacuation time of scheme B is close to that of scheme A.

**Figure 3:**
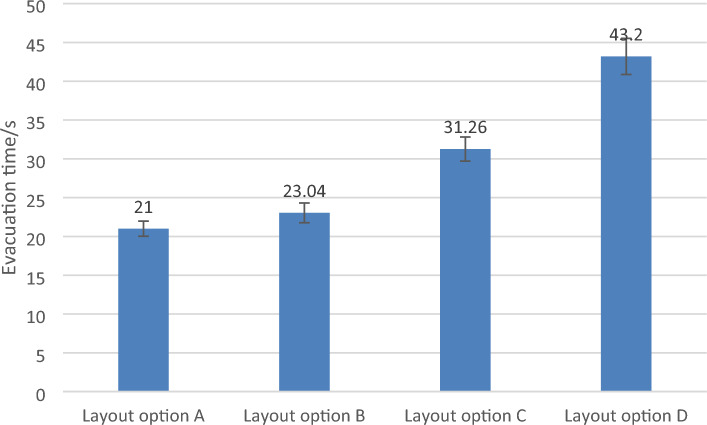
Evacuation time of four layout schemes.

SPSS24.0 software was used for data statistics and analysis. From the Levene Variance Homogeneity Test, the significance level was 0.221. It could be considered that the overall variance of the evacuation time of different layout schemes was homogeneous. Therefore, a one-way analysis of variance was performed. Results are shown in Table 4.

**Table 4:** Personnel walking speed distribution.

Levene statistics	1.541		Sum of squares	df	MS	F value	Significance
df1	3	Between groups	3112.308	3	1037.436	1226.284	0.000
df2	36	Within group	30.456	36	0.846		
Significance	0.221	Total	3142.764	39			

p < 0.05 indicates that the layout options have a significant impact on the evacuation time. From the perspective of improving evacuation efficiency and reducing evacuation time in critical situations, the layout option of the control center should be selected first, and the layout option A with the shortest evacuation time was selected first, followed by the overall layout option B.

**Figure 4:**
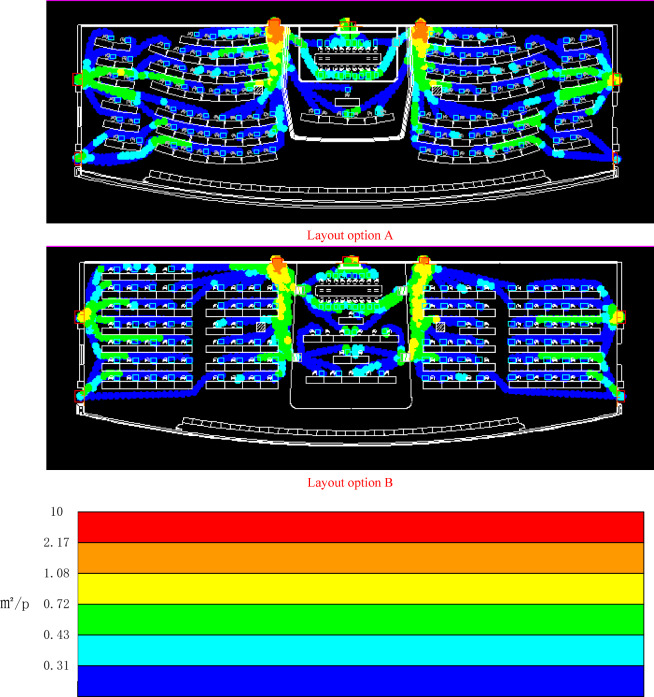
Example of cumulative maximum density map of layout option A & B in evacuation simulation.

When performing a detailed analysis, the cumulative maximum density map shows the maximum population density level in a specific area from the beginning of the simulation to the end of the simulation, which is used to measure the ability of the simulation analysis area to withstand specific standards. In this study, the cumulative maximum density map of the evacuation simulation of four layout schemes was used to analyze the pressure resistance level of different layout options, as examples shown in Fig. 4.

**Figure 5:**
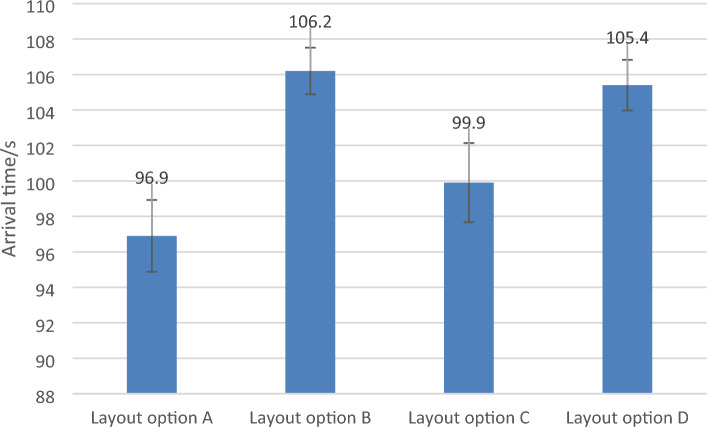
Arrival time of four different layout schemes.

Like the evacuation simulation analysis data processing steps, simulation analysis is performed on the control center personnel's arrival. The average arrival time of four layout schemes are shown in Fig. 5. It can be seen that among four layout schemes, the average arrival time of scheme A is the shortest, followed by scheme C.

One-way ANOVA is conducted for the simulation time of personnel's arrival in four layout schemes, and the results are shown in Table 5.

**Table 5:** Analysis of arrival time variance under different schemes.

Levene statistics	1.743		Sum of squares	df	MS	F value	Significance
df1	3	Between groups	595.80	3	198.60	61.741	0.000
df2	36	Within group	115.80	36	3.217		
Significance	0.176	Total	711.60	39			

p < 0.05 shows that different schemes have a significant impact on the simulation time of personnel post-arrival. The result verifies that Option A is the most preferred among the four options.

## Optimization for the ergonomics layout model

In the early 1960s, American scholar R. Muther proposed the Systematic Layout Planning (SLP)^29^. This method is widely used due to its clear logic and simple operation. The job layout problem can be solved by referring to the solution method of facility layout. This study draws on the ideas and methods of SLP. It introduces the human factors such as the personnel connection and the degree of association between each line and each post into the construction of the model and the determination of the objective function. The traditional SLP method is improved from the two aspects of modeling and solving algorithm to solve the problem of staff position layout.

### Simplification of job layout model

This study draws on the idea of rectangular simplification of layout space and facilities to be deployed in the two-dimensional Facility Layout Problem and the rectangular simplification of the control center's overall layout. The positions to be arranged in the control center are located in a simplified rectangular space with 7 rows and 18 columns. Sort the position of the console by serial number, a total of 116, each position corresponds to a dispatcher's position. In order to satisfy the COCC area's overall control of all positions and lines in the control center, the COCC area is arranged in the middle of the control center. The 5 scheduling seats of COCC are arranged according to user requirements, so they do not participate in the layout solution of the model built below. Therefore, the layout model has a total of 83 dispatchers, and the remaining 33 are reserved positions. The layout optimization problem is optimized for the position of the 83 dispatchers.

**Figure 6:**
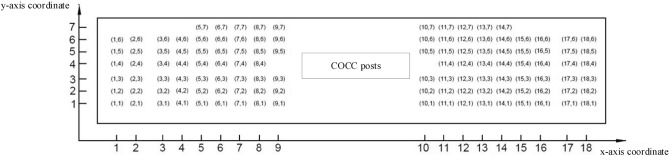
Control center layout simplified coordinates number diagram.

It should also be noted that considering the OCC standard line dispatcher, there is one seat between each row of adjacent two lines, for a total of 4 seats. After the layout optimization determines the positional relationship between the standard routes, the specific positions of the corresponding line dispatcher posts are also determined. Therefore, the layout optimization design variables do not consider the 4 seats of the standard line dispatcher. Since there is a preliminary layout plan for the control center, the ranks' coordinate number form is adopted when setting the position variables. Use a two-dimensional vector to express the discrete spatial position of each line post, as shown in Fig. 6.

### Construction of objective layout evaluation function

The objective function Eq. 1 is set as the degree of matching between the actual layout and ideal layout of each position in the control center. It is expressed by the variance between the actual layout relevance of each line post and the ideal relevance.

1 $$ \min z = \min \sum\limits_{i = 1}^{78} {\sum\limits_{j = i + 1}^{79} {z_{i \, j} } } $$

2 $$ z_{i \, j} = \left\{ \begin{array}{ll} 0 & \quad  g_{ij} = - 1 \wedge \frac{{d_{ij} }}{d} > 4 \hfill \\ \mathop {^{{\left[ {\left( {4 - \frac{{d_{ij} }}{d}} \right) - g_{ij} } \right]}} }\nolimits^{2} & \quad i,j \in \left\{ {{\text{Same}}\;{\text{Line}}} \right\} \hfill \\ \mathop {^{{2*\left[ {\left( {4 - \frac{{d_{ij} }}{d}} \right) - g_{ij} } \right]}} }\nolimits^{2} & \quad i,j \notin \left\{ {{\text{Same}}\;{\text{Line}}} \right\} \hfill \\ \end{array} \right. $$

Among them, g_ij_ = 4, 3, 2, 1, 0, − 1, which respectively represent the closeness of each post obtained by the SLP method; d is set as the average unit distance between adjacent posts, with the closeness between posts The relationship level is five. The farthest distance between the horizontal direction consoles of the control center divided by the number of levels is the value of d. From this, d = 14,980 is calculated; when the closeness of the relationship is -1, it means two. The positions cannot be close; that is, the distance value between the positions must be higher than 4d, so Z = 0 at this time. The minimum value of the sum of variances between the actual and ideal correlations between all functional positions is the initial layout plan that satisfies the relatively optimal relationship between them.

### Constraint of the correlation between various lines and jobs

To effectively play the role of coordination between the various line posts, considering the relevance between various line posts, the distance between the line posts with close operating relationships should be as small as possible to maximize the efficiency of dispatchers^30^. Among the control center posts, there were some special constraint requirements, for instance, the positions of all posts on the same line should be set in the same row. Some specific posts must be placed in fixed positions according to specific post functional requirements.

The spatial layout of the control center's lines and positions is affected by the closeness of the connections between the lines and positions. The closeness of the posts can be measured by the "relationship between the positions required to perform a task," and the closeness of the lines can be measured by the "relationship between the lines, the length of the line, and the degree of busyness." To quantify the closeness of the relationship is to express it in coefficient form.

Through questionnaires and expert interviews at the Shanghai Metro Control Center, combined with the analysis of the job functions and responsibilities of the control center personnel, we obtained the closeness relationship between each post and each line, as shown in Table 6 below. The relationship strength ranges from 4 to − 1. The larger the value, the stronger the relationship.

**Table 6:** Example of a standard line OCC posts interrelation.

	Traffic dispatcher 1	Traffic dispatcher 2	Traffic dispatcher 3	Integrated system dispatcher	Line dispatcher
Traffic dispatcher 1		4	4	3	4
Traffic dispatcher 2			4	3	4
Traffic dispatcher 3				3	4
Integrated system dispatcher					4
Line dispatcher					

### Determination of layout constraints

1. The uniqueness of job placement
  For each layout position, its capacity is 1, that is, each position has at most one post. The coordinate number between the two positions is different in at least one dimension, namely
  3 $$ x_{i} \ne x_{j} {\kern 1pt} \; \vee \;y_{i} \ne y_{j} \quad i \ne j,1 \le i,j \le 79 $$
  where (*x*_*i*_*,y*_*i*_) and (*x*_*j*_*,y*_*j*_) are the position coordinate numbers of post *i* and post *j* respectively.
2. Position constraints.
  - For each post of each line, the arrangement position of each post on the same line is restricted to be in the same row. Which is
    4 $$ y_{i} = y_{j} \quad i,j \in \left\{ {Same Line} \right\} $$
    Same as above, where *i* and *y* are the post number, and *y*_*i*_ and *y*_*j*_ are the coordinate numbers of post *i* and *j*, respectively.
  - For the standard route in the OCC area, the 3 traffic dispatcher of each route are required to be arranged adjacent to each other. The integrated system dispatcher of the line is arranged on the same side with the center line of the control center as the boundary. The specific mathematical expression is as follows:
    5 $$ \left\{ \begin{gathered} x_{i + 1} = x_{i} + 1 \hfill \\ x_{i + 2} = x_{i} + 2 \hfill \\ \end{gathered} \right. \quad  i = 1,5,9,13,...,41 $$
    6 $$ \left\{ \begin{array}{ll} x_{i + 3} = 1 \, \vee \, 2 & \quad { 3} \le x_{i} \le 7 \hfill \\ x_{i + 3} = 1{7} \vee {18} & \quad 10 \le x_{i} \le 14 \, \hfill \\ \end{array} \right. \quad i = 1,5,9,13,...,41 $$
    where *i* is the post number, *x*_*i*_, *x*_*i*+*1*_, *x*_*i*+*2*_ is the x coordinate number of three adjacent traffic dispatcher posts of a line, and *x*_*i*+*3*_ is the x-coordinate number of the integrated system dispatcher.
  - For fully automated operation lines, 4 traffic dispatchers, 1 line dispatcher, and 2 integrated system dispatchers are required to be arranged adjacent to each other. The specific mathematical expression is as follows:
    7 $$ \left\{ \begin{gathered} xi + 1 = 3 \, \vee \, 10 \hfill \\ xi + r = x_{i} + r \hfill \\ \end{gathered} \right. \quad i = 45,52,59,{\text{r}} = 1,2,...,6 $$
    where *i* is the post number, *r* is in the range of 1 ~ 6; *x*_*i*_ is the x coordinate number of post *i*, *x*_*i*+*r*_ is the x coordinate number of post *i* + *r.*
3. Post layout constraints.

A total of 7 network power dispatchers are required, and they are required to be arranged in the position space on the right side of the third row, which means that:

8 $$ \left\{ \begin{gathered} 10 \le x_{i} \le 18 \hfill \\ y_{i} = 3 \hfill \\ \end{gathered} \right. \quad 66 \le i \le 72 $$

In the same way, 7 network video dispatchers are required, and they are required to be arranged in the position space on the left side of the third row, that is, to meet:

9 $$ \left\{ \begin{gathered} 1 \le x_{i} \le 9 \hfill \\ y_{i} = 3 \hfill \\ \end{gathered} \right. \quad 73 \le i \le 79 $$

where *i* is the serial number of the post, 66 ≤ *i* ≤ 72 represents the serial number of the 7 network power dispatchers, 73 ≤ *i* ≤ 79 represents the serial number of the 7 network video dispatchers, (*x*_*i*_,*y*_*i*_) is the position coordinate number of post *i*.

## The solution of the optimal layout model

The improved genetic algorithm uses integer coding to genetically optimize the position coordinates of each line of the control center. A heuristic algorithm is designed to generate the initial population sequence that meets the constraints. Based on ensuring the validity of the initial population, the algorithm's optimization speed and quality are improved. To improve the operating efficiency of the genetic algorithm, an optimal preservation strategy is set. The elite individuals in the previous generation are used to replacing the worst individuals in the next generation to ensure the retention of good individuals and excellent genes in the population during evolution.

### Determination of integer coding

Since the genetic algorithm cannot directly operate on the established mathematical model of the control center layout optimization, we need to convert the solution of the model into a chromosome or individual composed of genes in a specific sequence structure in the genetic space. This conversion process is called encoding. In this algorithm, the gene is defined as the position coordinates of each post of the control center. That is, the smallest unit of the model solution is a specific post of the control center. The minimum unit is not set as the OCC of a line: (1) It is more flexible in solving each position as the minimum unit of the model. When the OCC of a line is not in the same area, the control The layout of the center is more adaptable. (2) If the smallest unit is defined as an OCC line since the order of posts in a line is fixed, it is difficult to meet the requirements when the degree of correlation between OCC posts on different lines is optimized. (3) In the objective function built in this paper, d is the average unit distance between adjacent positions. The value of d is unique and easy to calculate. If the smallest unit is an OCC line, the value of d is not unique and difficult to calculate. Therefore, this article sets a single post as the smallest unit of the model.

**Figure 7:**
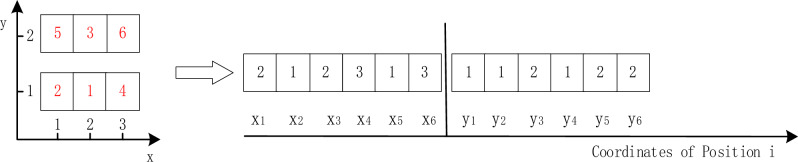
Integer coding for line positions.

In this paper, integer coding is used, and an overall layout plan is an individual composed of multiple genes. Assuming that the control center has six positions to be arranged, the code of each individual is a 1-dimensional vector containing 12 values. The first six represent the X coordinate number of each post, and the last six represent the Y coordinate number of each post. The coding method is shown in Fig. 7. The figure on the left shows the manifestation of an individual, that is, a layout scheme. The right figure shows the corresponding genotype, that is, the form of individual operation in genetic algorithm.

### Population initialization based on heuristic algorithm

The genetic algorithm is an optimization operation for the evolution of the population, and the initial population that represents the starting search point needs to be prepared for it. At present, it is widely used to initialize the overall population by randomly generating a set of feasible solutions, but the method of randomly initializing the population cannot guarantee the rationality of the population and the efficiency of the algorithm. Aiming at the numerous constraints on the job layout of the Shanghai Metro Control Center and the design variables with repeated values, this paper designs a heuristic algorithm to generate an initial population that meets the constraints and improves the algorithm's efficiency for subsequent iterations. Seek the best as the foundation. Here, the previous design variables are simplified, taking the arrangement of seven positions as an example to describe the heuristic algorithm process.

- Step 1. Define p1 as an individual in the initial population and set its initial value to be 0.
- Step 2. Determine the specific layout positions of some posts. According to the previous constraint settings, first determine the posts that require fixed positions, as well as the posts that need to be arranged continuously, such as the fully automatic driving line, and set them as the first four posts.
- Step 3. Determine the specific location of the remaining posts. Based on the previous layout, the remaining arranging space can be obtained. The remaining arranging space can be randomly selected to arrange the remaining posts. According to the previous constraint settings, determine such as standard route line coordination and integration The layout position of the transfer, the layout position of the comprehensive transfer is arranged on the same side of the line with the centerline of the control center as transfer post of the line.

### Determination of fitness function of the improved genetic algorithm and design of genetic operator

The fitness function of the genetic algorithm is used to evaluate the pros and cons of individuals in the population and is usually calibrated according to the objective function of the problem. We directly use the objective function of the above layout optimization mathematical model as the fitness function and minimize the objective function as the optimization objective for genetic optimization. For the treatment of the uniqueness principle of job placement, the penalty function form is adopted. The objective function increases by a significant positive value. Therefore, the calculation formula of d is as follows.

10 $$ \begin{aligned} d_{ij} = & |(X_{i} - X_{j} )| + |Y_{i} - Y_{j} | \\ X_{i} = & f(x_{i} ) = \left\{ {\begin{array}{*{20}l} {1100 + 2800x_{i} \, } \hfill & {1 \le x_{i} \le 2} \hfill \\ {3100 + 2800x_{i} \, } \hfill & {3 \le x_{i} \le 9} \hfill \\ {26,400 + 2800x_{i} } \hfill & {10 \le x_{i} \le 16} \hfill \\ {28,400 + 2800x_{i} } \hfill & {17 \le x_{i} \le 18} \hfill \\ \end{array} } \right. \\ \, Y_{i} = & h(y_{i} ) = \left\{ {\begin{array}{*{20}l} {3200 + 2600y_{i} } \hfill & {1 \le y_{i} \le 2} \hfill \\ { \, 11,500 \, } \hfill & { \, y_{i} = 3} \hfill \\ {4200 + 2600y_{i} } \hfill & {4 \le y_{i} \le 7} \hfill \\ \end{array} } \right. \\ \end{aligned} $$

The design of "selection operator". Selection is the operation of selecting individuals with good fitness from the group and eliminating poor individuals. It aims to select better individuals to generate new individuals through pairing, crossover, mutation, or directly inherited to the next generation. We use the roulette selection operator, which is a ratio-based selection method. Since we use the fitness function of minimizing the optimization goal as the genetic operation, the smaller the fitness function, the better the target individual, which is contrary to roulette selecting the individual with the greatest fitness. Therefore, before the selection operation, the fitness function of each individual needs to be transformed.

The design of "Cross operator". Crossover refers to the operation of replacing and recombining part of the genetic structure of two-parent individuals to generate new individuals. We adopt a crossover method based on the single cut point of the bound genome and perform repeated gene repair on the individuals after the crossover.

The design of "Mutation operator". To enhance the local search capability of the genetic algorithm and maintain the diversity of the population, mutation operators need to be added to the algorithm. The main content of mutation operation is to make partial changes to some gene values on individual gene positions in the population. We adopt the interchange mutation method based on bound genome.

### Validation of improved genetic algorithm and determination of layout plan

The Python software platform is used to realize the optimization solution process of the above improved genetic algorithm. Aiming at the position layout problem of the control center in this study, the population size of the designed improved genetic algorithm is fixed to 10, and the genetic algebra is set to 50 generations. Run 10 times under different cross-mutation combinations to find the average fitness of each combination. Finally, the crossover rate P_c_ = 0.7 and the mutation rate P_m_ = 0.5 are the best crossover mutation combinations for the improved genetic algorithm.

In order to verify the effectiveness of the improved genetic algorithm proposed in this paper, first, by fixing the probability of crossover and mutation and running the algorithm program independently, the best combination of crossover rate and mutation rate is obtained. Under this combination, the improved genetic algorithm can be compared with high probability to find the optimal solution. Under the same conditions of population number and iteration number, the combination of crossover and mutation rate is used as the genetic parameter setting of the improved genetic algorithm, combined with other settings. The solution results are compared to further verify the effectiveness of the proposed algorithm and the efficiency of the solution process.

Under the same population size and number of iterations, the two genetic algorithms are run 10 times, respectively, and the optimal fitness value and average fitness value of the two are compared. The results are shown in Table 7. The algorithm stopping criterion is that the genetic algebra k reaches the maximum upper limit G_max_. Using SPSS24.0 to test the fitness calculated by the two methods, there is a significant difference in the mean t-test results (t = − 89.153, df = 9.818, Sig = 0.000).

**Table 7:** Comparison of two algorithms.

	N	Means	Best	Std
Improved genetic algorithm	10	26,983	26,359	451.61
Global optimization toolbox in MATLAB	10	87,983	84,502	2116.03

**Figure 8:**
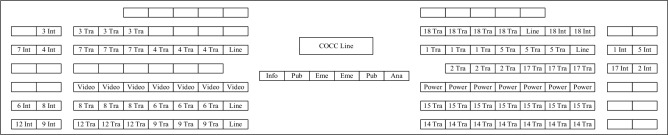
Visual result of posts layout diagram. Int refers to comprehensive dispatch, Tra refers to traffic dispatch, Line refers to dispatch chief, Video refers to network video monitoring dispatch, Power refers to network power dispatch, Info refers to information dispatch, Pub refers to public dispatch, Eme refers to emergency dispatch and Ana refers to analysis dispatch.

It proves the effectiveness of the proposed improved genetic algorithm in solving the job layout problem of the control center. The optimal solution was visualized, as shown in Fig. 8.

## Discussion

This research takes the metro control center as the research object. According to task requirements and relevant control center human factors engineering standards, a human factors-based control center layout design method that meets the requirements of metro operations is proposed. Targeting the layout of the Shanghai Metro Control Center project under construction been studied in detail. Different from the emphasis of Naito et al., due to the particularity of the control center, this study embeds the correlation analysis in different work scenarios^6^. Based on Quintana and Aas focusing on petrochemicals, and Duarte focusing on maritime^13^, this study further broadens the control center's human factors design range.

The layout design between the consoles of different positions in the control center uses functional connections to arrange multiple workstations in the practical space of the control center and convert functional groups into workstations of appropriate size. And it's carefully adjusted to ensure all space for working, circulation, and maintenance of personnel and equipment^31^. Similar to traditional research methods, this research also considers the total area of the hall. On this basis, in accordance with the characteristics of the subway control center, factors such as the number of lines, the number of posts per line, the closeness of connections between lines and posts within each line are introduced into the layout design of the console. When determining the single-person space, research is carried out through the single-post console's design ideas and methods. The calculation of personal space requirements based on task requirements is more accurate than the original way. Based on combining the hard constraints of human factors standards, and comprehensively considering the different needs of different positions, personnel, and job types for display control equipment, the three-dimensional space dimensions obtained are more accurate and economical.

The SLP method's design process requires cumbersome iterative operations and is subject to subjective influences, resulting in unstable results. The genetic algorithm used in this study is robust and intelligent^32^. On the basis of the preliminary overall layout plan of the control center, an in-depth discussion was carried out on the optimized layout of 79 different professional positions. Based on the principle of SLP facility layout, an optimized mathematical model based on the correlation of each position and the correlation of different subway lines was established. The results obtained in this research are mutually confirmed by applying genetic algorithms in traditional workshops^33,34^, and provide references for solving large-scale complex facility layout problems^35^.

This method is based on simplifying the rectangular space model of the control center for layout optimization design. The actual internal space of the control center is changeable. Therefore, the layout of the complicated control center with irregular space needs further research. In constructing the objective function for job optimization, this article only considers the close correlation between different routes and between different positions^36^. Later, more relevant factors such as line length and line busyness can be integrated into the objective function to make the objective function more in line with the control center's actual operational needs. The model solving algorithm designed in this paper has strong pertinence and is only suitable for solving the same type of model. Due to the variability of the layout model in terms of the objective function and layout constraints, it is necessary to make further improvements in algorithm parameters and process design for more complex layout optimization problems.

## Conclusion

The overall effective operation of the control center depends on the reasonable cooperation of all personnel and equipment. This research summarizes the workflow of the control center layout design process. Starting from the difference in the requirements of each operator for different equipment, determine the difference in space requirements of different positions, and obtain different physical space units. The location and space planning are carried out through the physical space constraints of relevant standards, and then the discrete simulation of personnel mobility is used to optimize the plan. Finally, combining the connections between the positions and the specific constraints, the specific personnel are arranged in specific positions to complete the control center's position layout. Based on the layout results of this research, the post layout plan of the Shanghai Metro Control Center has been optimized, which also provides a positive reference for the subway planning and design of other cities. In particular, this article shows the importance of ergonomics in the layout design of the subway control center. In a broader sense, since this research represents the practical application of the combination of ergonomic principles and intelligent algorithms, it can also be used as a reference for research on similar equipment and facilities.

## Data Availability

The datasets used and/or analyzed during the current study are available from the corresponding author on reasonable request.
